# Exploring the bone marrow micro environment in thalassemia patients: potential therapeutic alternatives

**DOI:** 10.3389/fimmu.2024.1403458

**Published:** 2024-08-05

**Authors:** Zengzheng Li, Xiangmei Yao, Jie Zhang, Jinghui Yang, Junxue Ni, Yajie Wang

**Affiliations:** ^1^ Department of Hematology, The First People’s Hospital of Yunnan Province, The Affiliated Hospital of Kunming University of Science and Technology, Kunming, Yunnan, China; ^2^ Yunnan Province Clinical Research Center for Hematologic Disease, The First People’s Hospital of Yunnan Province, Kunming, Yunnan, China; ^3^ Yunnan Provincial Clinical Medical Center for Blood Diseases and Thrombosis Prevention and Treatment, Kunming, Yunnan, China; ^4^ Department of Medical Genetics, The First People’s Hospital of Yunnan Province, The Affiliated Hospital of Kunming University of Science and Technology, Kunming, Yunnan, China; ^5^ Department of Pediatrics, The First People’s Hospital of Yunnan Province, The Affiliated Hospital of Kunming University of Science and Technology, Kunming, Yunnan, China; ^6^ Hospital Office, The First People’s Hospital of Yunnan Province, Kunming, Yunnan, China

**Keywords:** beta-thalassemia, osteoblast, impaired hematopoiesis, metabolic abnormalities, ineffective erythropoiesis, microenvironment correction

## Abstract

Genetic mutations in the β-globin gene lead to a decrease or removal of the β-globin chain, causing the build-up of unstable alpha-hemoglobin. This condition is referred to as beta-thalassemia (BT). The present treatment strategies primarily target the correction of defective erythropoiesis, with a particular emphasis on gene therapy and hematopoietic stem cell transplantation. However, the presence of inefficient erythropoiesis in BT bone marrow (BM) is likely to disturb the previously functioning BM microenvironment. This includes accumulation of various macromolecules, damage to hematopoietic function, destruction of bone cell production and damage to osteoblast(OBs), and so on. In addition, the changes of BT BM microenvironment may have a certain correlation with the occurrence of hematological malignancies. Correction of the microenvironment can be achieved through treatments such as iron chelation, antioxidants, hypoglycemia, and biologics. Hence, This review describes damage in the BT BM microenvironment and some potential remedies.

## 1 Introduction

BT is an autosomal recessive hematological condition characterized by an imbalance between α-globin and β-globin chains, leading to inefficient erythropoiesis (1, 2). BT can cause iron overload (IO), extramedullary hematopoiesis, BM expansion, hemolytic anemia, and multiple organ involvement, which has a significant clinical impact on patients (2–4). In BT patients, the unbound α-globin binds to free heme molecules to form toxic insoluble aggregates (called hemipigment), which precipitate and destroy the red blood cell (RBC) membrane. Simultaneously, they also initiate the production of reactive oxygen species (ROS), leading to oxidative stress and impacting the longevity of specific subsets of RBC (5, 6). Heat shock protein 70 (HSP70) is consistently expressed in human erythroblasts. As these erythroblasts mature, HSP70 moves into the nucleus and protects GATA-1, the primary transcription factor responsible for erythrocyte production, by preventing caspase-3 cleavage (7). An excess of free α-globin chains binds to HSP70, leaving GATA-1 unprotected which in turn causes cleavage and degradation by caspase-3. Ultimately, end-stage maturation arrest and erythroid progenitor cell apoptosis further impair RBC production (7). Additionally, ROS facilitates the excessive production of growth differentiation factor 11 (GDF11) in BT. This excessive production of GDF11 hinders the process of erythropoiesis in BT by activating SMAD2/3 signal transduction, which is involved in controlling the differentiation of RBC. As a result, the differentiation of erythrocytes is restricted (8, 9). It also promoted erythroid amplification and ineffective erythrocyte production in BT (8). This series of events leads to early apoptosis of mature nucleated erythrocytes, accompanied by hematopoietic amplification, followed by chronic hemolytic anemia with significant reticulocytosis, severe anemia, and a series of secondary pathophysiological mechanisms (10).

Heterogeneous populations of stromal cells and extracellular matrix form a specific microenvironment in the BM (11). The hematopoietic stem cells found in the BM establish a dynamic relationship with the surrounding microenvironment, ensuring the equilibrium of the body’s hematopoietic system (12). However, the BM microenvironment changes under some pathological conditions (13). For example, the tumor cells of acute myeloid leukemia are derived from the malignant transformation of hematopoietic stem cells and are able to alter the microenvironment, allowing the BM microenvironment to develop into a more suitable tumor microenvironment for tumor cell growth (14). This provides a refuge for malignant cells, allowing them to enter a chemotherapy-resistant state and become more prone to recurrence (15, 16). Unlike this, BT is a change in the BM microenvironment caused by ineffective erythrocyte production (17). This may not provide benefits for ineffective erythropoiesis, but rather consistently damage the microenvironment. This review focuses on the possible correlations between the accumulation of macromolecules in the BM, impaired maintenance of hematopoietic function, disruption of bone cell differentiation, destruction of bone structure, thalassemia, and hematologic tumors. Furthermore, possible approaches that might potentially provide therapeutic benefits are also discussed. This may aid in comprehending and managing the BM microenvironment of BT.

## 2 Bone marrow microevent and cellular composition and lineage in normal condition

Cell proliferation and BM activity are increased in the BM of patients with BT during inefficient erythropoiesis, despite the fact that the process is not functional, it is not completely halted (18). Attenuated Total Reflection-Fourier Transform Infrared Spectroscopy (ATR-FTIR) found that lipid, protein, glycogen, and nucleic acid contents in thalassemia BM mesenchymal stem cells (MSCs) were significantly higher than those in normal MSCs (19). The content of these macromolecules in BM MSCs of BT patients after hematopoietic stem cell transplantation was significantly lower than that before transplantation (19). This indicate that hematopoietic stem cell transplantation can effectively address the accumulation of macromolecules in MSCs. Furthermore, the degree of fat unsaturation in the BM increases proportionally with the increase in erythropoiesis (20). In patients with BT, the amount of fat and apolipoprotein D (APOD) in the BM decreases, but the levels of unsaturated fatty acids(UFA) increase. This phenomenon is associated with ineffective erythropoiesis (21, 22). Studies have shown that hydroxyurea therapy can improve the abnormal metabolic pathways of lipoprotein changes, glycolysis, Tricarboxylic acid cycle, fatty acid, and choline metabolism in BT patients (23–25). Additionally, individuals with transfusion-dependent thalassemia (TDT) commonly experience diabetic mellitus (DM) as a consequence, and there is a significant accumulation of glycogen in the BM microenvironment (19, 26). Patients with thalassemia need to be provided with appropriate treatment and monitored for a long time. Some patients develop diabetes even after hematopoietic stem cell transplantation (27). Oral hypoglycemic agents are effective and safe in the treatment of DM in TDT patients and can achieve adequate blood glucose control in a considerable time (28). Due to the accumulation of glycogen in the BM microenvironment, long-term blood glucose monitoring and appropriate treatment should be given to patients with thalassemia. Metformin is an oral hypoglycemic drug with multiple effects. Additionally, it can help preserve the integrity of DNA (29). It has a beneficial impact on cardiac function and reduces the chances of heart failure and renal damage (30, 31). Engliflozin (Em) also has the effect of controlling blood sugar and can reverse the PlGF-1 resistance phenotype of hyperglycemic monocytes. Moreover, Em also can restore EC dysfunction in hyperglycemia, which may be attributed to the recovery of VEGFR-2 receptors on the EC surface (32). Most SGLT-1 and 2 inhibitors, including Sotagliflozin, have good effects on improving ROS, hyperglycemia, EC dysfunction, and heart failure (33, 34).

In the BM of BT patients, excessive α-globin chain accumulates in the progenitor RBC, resulting in the premature death of the progenitor RBC in the middle and late stage of BM, further aggravating the increase of the levels of erythropoietin (EPO) and growth differentiation factor 15 (GDF-15) (35). Furthermore, the levels of ferritin in the BM plasma of patients with BT were markedly elevated compared to both normal people and BT patients who had undergone transplantation, regardless of their usage of iron chelators (36–38). At the same time, the content of ferritin in the BM plasma of BT was also significantly higher than that in peripheral blood (39). This evidence indicates that there is a large amount of iron accumulation in the BM of BT patients. An important explanation for this outcome is that MSCs can take up iron through ferroportin (For example SLC40A1, a protective mechanism against accumulation of cytoplasmic free iron) and transferrin receptor 1 (TFR1), and express the ferritin gene to store iron (40). This may be a protective measure against IO. However, prolonged exposure to iron can change MSCs’ systems for sensing and storing iron, which is primarily shown by their failure to stimulate SLC40A1 expression. This can lead to a buildup of cytoplasmic free iron (40, 41). Increased ROS levels will accompany iron accumulation in transfusion-independent patients, and oxidative damage from ROS is a major factor in thalassemia patients’ cell and tissue damage (42). In recent studies, it has been observed that the production of ROS can also occur through the action of cytochrome P450 (CYP450) 4A and 4F, which induce the production of 20-hydroxyeicosatetraenoic acid (20-HETE) (43). This overproduction of ROS was observed in *Hbb^th3/+^* mice “model of β-thalassemia” and was found to be mediated by a pathway that is dependent on nicotinamide adenine dinucleotide phosphate (NADPH) (43, 44). Further increases in ROS levels in BT-MSCs resulted in decreased expression of antioxidant genes, altered ferroportin activation, and inappropriate regulation of iron-related genes such as TFR1 in BT-MSCs (42, 45, 46). The iron-overloaded BM environment impairs iron-sensing mechanisms, which may generate oxidative stress and alter the functional properties of MSCs (47, 48).

The accumulation of iron poses great harm to the BM microenvironment, leading to a series of deleterious effects including ROS. Iron chelation therapy is advantageous for ameliorating impaired lung function, renal function, cardiac function, vascular function, endocrine function, ROS, and ROS-induced chain reactions resulting from IO (49–51). At present, the most frequently used drugs for iron chelation in thalassemia are deferoxamine (DFO), deferiprone (DFP), and deferasirox (DFX), which can reduce the incidence rate and mortality related to organ iron deposition, including BT patients with Hematopoietic stem cell transplantation (52). It has been seen that DFX has good safety and controllability when used in therapy (53–55). Additionally, the approved luspatercept is an erythroid maturation agent that can be combined with selected transforming growth factor beta (TGF-β) superfamily ligands to reduce Smad 2/3 signaling and enhance late-stage erythropoiesis. Adult patients diagnosed with BT major (β-TM) now have a novel therapy available for long-term management (56). This medication seeks to reduce the necessity for frequent RBC transfusions, decrease anemia, and avoid excessive iron accumulation (56). Ferritin agonists can not only improve IO but also manage intermediate β-Anemia and liver burden in patients with thalassemia (57).

## 3 Bone micro environment in thalassemia patient

### 3.1 Hematopoietic stem cell quiescent state loss

Compared with normal mice, hematopoietic stem cells(HSCs) from *Hbb^th3/+^* mice lose their quiescent state and enter the cell cycle. The primary observation is that the frequency of HSCs in the G0/G1 phase is significantly diminished, while the proportion of cells in the S phase increases and the number of colony-forming units is reduced (17, 58). Transplantation of HSCs derived from *Hbb^th3/+^* mice into normal mice restored the long-term repopulation capacity of HSCs, whereas HSCs from normal mice transplanted into *Hbb^th3/+^* mice inhibited the reconstitution process (17). These pieces of evidence are adequate to indicate that the function of HSCs is compromised in the myeloid environment of BT. This design provides more evidence that the BM microenvironment of the BT has an impact on the biological activity of HSCs. Due to chronically active ROS, patients with transfusion-dependent BT require higher stimulation of the CD34^+^ response to stress and have higher circulating rates of primitive hematopoietic stem progenitor cells (HSPCs) (39). Single-cell sequencing approaches revealed a higher percentage of CD34^+^ B lymphoid progenitors and a lower percentage of other stem and progenitor cell types in the CD34 compartment in pediatric BT patients (59). Studies have shown that daily doses of recombinant parathyroid hormone (PTH) can rescue HSCs defects by restoring the expression of stem cell genes (17). The main reason was that after *Hbb^th3/+^* mice received PTH treatment, the number of resting HSCs increased and the level of cyclin-dependent protein kinase inhibitor 1C (CDKN1C) was also recovered (17, 60).

MSCs have been used *in vitro* to support the expansion of HSCs and HSPCs, and *in vivo* to promote HSPCs implantation (61). BM-MSCs are also damaged in BT patients, so will HSCs transplantation with BM-MSCs improve transplant outcomes? Clinical studies have found that the use of combined BM-derived MSCs does not affect the transplant outcome of type III thalassemia (62, 63). However, it has been found that bioactive molecules in extracellular vesicles derived from MSCS can regulate the expression of HSCs genes *BIRC34*, *BIRC2*, and *NF-κB* to improve the cloning ability of CD34^+^ cells (64–66). However, the practical use of exosomes for treatment has not yet achieved any significant advancements.

### 3.2 Impaired MSCs function

The preservation of hematopoietic function is closely linked to the biological role of MSCs. Recent research has revealed a decrease in the occurrence of CD146^+^ and CD271^+^ cells in the BM of BT patients, and this decrease is inversely associated with levels of ROS (17). Moreover, the expression frequencies of CD73 and Sca-1 in MSCs of BM of *Hbb^th3/+^* mice were also found to be decreased (17). The reduced levels of KIT ligand (KITLG) and CXC chemokine ligand 12 (CXCL12), which are crucial molecules for the maintenance of hematopoiesis, hindered the implantation, retention, survival, and proliferation of HSCs, are also observed (39, 67). Angiopoietin-1 (ANGPT1) and vascular endothelial growth factor (VEGFA), which regulate the quiescence of HSPC, were inhibited as well (39, 68). Fibroblast growth factor 2 (FGF2) and interleukin 6 (IL-6), which have amplification effects on HSPC and paracrine proliferation effects on MSC, are also inhibited (39, 69). Additionally, the amount of CD34^+^ attracted by BT MSCs is much lower compared to normal BM-MSCs, and its effect in promoting the expansion of umbilical cord blood HSCs is also poor (17, 39, 70). Inhibition of these molecules may be one of the reasons for the poor efficacy of BT HSCs transplantation.

The ability of CD105^+^ MSCs obtained from the BM of BT patients to differentiate into osteogenic cells is significantly limited (71). Runt-related transcription factor 2 (RUNX2), which is efficiently activated in normal MSCs, is a key factor regulating osteocyte production (72, 73). The expression of RUNX2 in MSCs was seen to be decreased in BT, leading to a downregulation of the secreted protein acidic and rich in cysteine (SPARC) and collagen type I alpha 2 (COL1A2) (39). Consequently, the formation of mineralized bone is hindered (39). The subchondral trabecular network density, bone mineral density, and trabecular number were decreased in the BM of *Hbb^th3/+^* mice while the distance between trabeculae is enlarged (17, 74). This indicates that the process of MSCs transforming into OBs in the BM environment of patients with BT is impeded. Compared with the normal BM microenvironment, the expression of Notch ligand Jagged 1 is reduced in the BT mouse microenvironment (17). Loss of Jagged1 favors the induction of OBs ablation (17, 75). In a study, the formation of MSC-derived bone structures of BT in a humanized ossicle mouse model was significantly delayed (39, 76). The main manifestations were reduced bone-cell formation, hollow bone cavity, reduced number of blood vessels, and the formation of immature bone and abundant extracellular matrix. Moreover, few hematopoietic cells colonized, consistent with the impaired maintenance of hematopoiesis described above (39). The methylation of histone 3 lysine 9 (H3K9) and histone 3 lysine 36 (H3K36) was significantly downregulated in iron-overloaded BT-BM MSCs. This suggests an altered ability of MSCs to form appropriate niches *in vivo*.

### 3.3 Impaired OBs formation

The first unsuccessful erythropoiesis in thalassemia patients results in BM enlargement, which lowers trabecular bone tissue and thins the cortical layer. Second, endocrine disruption brought on by high iron loading results in higher bone turnover (77). MSCs derived from the BM of BT patients have weakened cloning ability, low proliferation rate, and inefficient differentiation ability, so the MSCs in the BM of BT cannot effectively differentiate into OBs *in vivo*. Moreover, the same bones are formed as the bone disease in BT patients (39). Further development includes osteoporosis, growth failure, spinal deformity, and fragility fracture diseases (78, 79). This is a significant factor contributing to the prevalence of bone-related diseases in BT patients (78, 79). Reduced OBs activity is the primary cause of low bone mineral density, and it may not be associated with osteoclast (OC) activity (17). The levels of alkaline phosphatase (ALP), an indicator of OBs activity, and the bone matrix glycoprotein osteopontin(OPN), mostly synthesized by OBs, were decreased in the *Hbb^th3/+^* mice. Additionally, the absence of OPN resulted in HSCs progression into the cell cycle (80). Studies have shown that PTH can rescue HSCs function by increasing OPN levels (60). In thalassemia patients, the primary impairment of osteocyte lineage cells is OBs.

OBs themselves express transferrin receptor (TFR) and divalent metal transporter 1 (DMT1) (81). In the iron-overloaded BM microenvironment, iron toxicity can affect OBs to undergo apoptosis by directly altering bone microarchitecture or inducing oxidative stress (82, 83). Previous *in vitro* studies have shown that increased levels of ROS caused by IO have a severe impact on OBs proliferation, autophagy occurrence, differentiation, and mineralization in human, murine, and osteoblast-like cells (40, 84, 85). ROS may function by suppressing the PI3K/Akt/mTOR pathway, resulting in the stimulation of glycogen synthase kinase 3β (GSK-3β) (86, 87). Additionally, IO reduces canonical Wnt signaling, which further activates GSK-3β. The phosphorylation of GSK-3β is essential for the process of bone formation (osteogenesis). It directly controls the activity of RUNX2 or indirectly prevents the breakdown of β-catenin (88, 89). As a regulator of OBs differentiation and formation, Forkhead box transcription factor 1 (FOXO1) can directly interact with the promoter of RUNX2 to regulate its expression (90). FOX1 promotes bone formation by decreasing oxidative stress in OBs. ROS generated by IO may activate PI3K/Akt, leading to the inhibition of FOXO1 expression. This, in turn, impairs the survival of OBs (91, 92).

In addition, high levels of erythropoietin can directly affect the differentiation and mineralization of OBs progenitors, resulting in decreased bone density (93). This could be attributed to the overproduction of fibroblast growth factor (FGF)-5 in bone and BM erythroid cells, stimulated by erythropoietin through ERK23/1 and STAT2 pathways (94). Consequently, this results in the upregulation of FGF23 expression in bone and BM erythroid cells, leading to increased levels in BT patients and mice. Ultimately, this results in alterations in bone mineralization and disposition (94, 95). Inhibiting FGF23 signaling through carboxyl terminated FGF23 peptide is a safe and efficacious therapeutic approach to rescue bone mineralization and deposition in mice with β-thalassemia, normalizing the expression of niche factors and restoring HSCs function (94, 96). Additionally, OBs exposed to hyperglycemia show impaired function, such as decreased expression of ALP, GLA proteins, and OPN (97–99).

Bone resorption, a common complication of thalassemia, has been shown to increase bone mineral density (BMD) in the femoral neck, lumbar spine, and forearm after two years of bisphosphonate treatment. After 12 months, the addition of zinc to one’s diet may lead to an increase in BMD specifically in the lumbar spine and hips (100). Moreover, supplementing zinc may improve iron-induced pancreatic exocrine and endocrine dysfunction (101). Current modalities for the management of osteoporosis in adults with TDT include inhibitors of bone remodeling such as bisphosphonates and denosumab as well as stimulators of bone formation, like teriparatide (28).

## 4 Bone microenvironment in case of bone marrow transplantation (BMT) patient

Poor transplantation function is a life-threatening complication that occurs after transplantation and has poor prognosis, limiting the success of BMT. The damaged bone marrow microenvironment is one of the important causes (102). In addition to the damage to the microenvironment itself, the treatment scheme used for transplantation can also damage and exacerbate the damage to the microenvironment. Cyclophosphamide(Cy) chemotherapy altered survival or proliferation of growth plate chondrocytes and metaphyseal osteoblastic cells and reduced heights of metaphyseal spongiosa trabecular bone, which may contribute to chemotherapy side effects of this drug on bone lengthening and bone mass accumulation (103). Cy reduces the number and differentiation of bone mesenchymal stem cells, as well as the formation and activity of osteoblasts. In addition, Cy inhibits the formation of osteoclasts by reducing their maturation and activity (104). Bone resorption is a common complication in thalassemia, and bisphosphonates may increase bone mineral density (BMD) in the femoral neck, lumbar spine, and forearm after two years of treatment. Zinc supplementation may increase BMD in the lumbar spine and hips after 12 months (100). In addition, supplementing zinc may improve iron induced pancreatic exocrine and endocrine dysfunction (101). Current modalities for the management of osteoporosis in adults with TDT include inhibitors of bone remodeling such as bisphosphonates and denosumab as well as stimulators of bone formation (e.g., teriparatide) (28). In addition, the myeloablative regimen can cause damage to BM EC, and the hematopoietic defects caused by damaged BM EC are positively correlated with ROS levels (105).

According to reports, atorvastatin is a widely used lipid-lowering drug in clinical practice, which improves the functional impairment of BM EPC in the body by downregulating the p38 MAPK pathway. In addition, NAC can reduce ROS levels *in vitro* and *in vivo*, while repairing damaged BM ECs to effectively promote hematopoietic reconstruction (105, 106). Due to the damage caused by Busulfan(BU) and Cy, researchers are constantly developing new protocols for pre-transplantation injection of BU or trisulfan (TREO) combined with thiacloprid (TT) and fludarabine (FLU) in patients with thalassemia, which have better overall survival and event free survival while reducing transplant failure (107). In addition, the combination of CY, intravenous BU, FLU, TT, and ATG (named NF-08-TM) has achieved excellent results in Mediterranean transplantation (108, 109). The RIC regimen combined with hydroxyurea (HU), alemtuzumab, FLU, melfaram (MEL), and TT is also superior to the BU-CY regimen (110). You can also add BIRC34, BIRC2, and NF- κ B expression to increase the cloning ability of CD34 cells.

## 5 Overall bone marrow pathological condition and scope of drug target based on BM condition

ROS is involved in the impaired function of MSCs, HSCs, OBs formation, and a series of complications in the BM microenvironment, which may be the second major factor affecting the BM microenvironment. The excess ROS can be neutralized by an efficient antioxidant system, which includes antioxidant enzymes and non-enzymatic molecules (111). Common antioxidant enzymes include superoxide dismutase (SOD), catalase, peroxidase (PRDXs), peroxiredoxin (Prxs), and glutathione peroxidase (GPXs) (111). However, the mechanism is inadequate in regulating ROS to reach normal levels in the injured BM microenvironment. At present, it can be regulated through other non-enzymatic antioxidant molecules such as glutathione, flavonoids, thioredoxin, and vitamins A, C, and E. These substances can be readily acquired as they are frequently found in plants (112). Resveratrol, found in the skin of fruits including grapes, blueberries, and raspberries, is a potent scavenger of ROS. It exerts a defensive influence on lipid peroxidation occurring in the plasma membrane and guards against DNA damage induced by ROS (113). Quercetin is a polyphenolic flavonoid found in apples, radishes, coriander, and cranberries, which can scavenge ROS and can be expressed through MAPK/ERK1/2, JAK/STAT and TRAIL, AMPK α 1/ASK1/p38, RAGE/PI3K/AKT/mTOR axis, HMGB1, and NF- κ B. Nrf2 and other signaling pathways regulate cell state (114). N-acetylcysteine (NAC) is an antioxidant derived from a small amino acid with a low molecular weight, which allows it to be rapidly delivered to the cytoplasm (115). Moreover, NAC has been shown to greatly enhance bone healing and its ability to promote bone formation is evident. However, further research is required to fully understand the particular mechanism behind this benefit (115). Other sources of dietary antioxidants include rutin, anthocyanins, chlorogenic acid, quinic acid, caffeic acid, etc (116). These substances all regulate the activity of endogenous oxidase systems and their related proteins, preventing oxidative damage to organelles, proteins, nucleic acids, and lipids (117). Although there is evidence to support these effects at this time, to avoid adverse reactions, it is important to consider the dose when using these substances. Patients with thalassemia should be advised to add foods high in these compounds to their diet in addition to receiving therapy.

## 6 Thalassemia and hematological tumors

In recent years, with the continuous improvement of treatment plans, the survival rate of patients with thalassemia has been significantly improved. However, more complications have emerged, such as the rising incidence of liver cancer (118). A comprehensive longitudinal research conducted in Taiwan has revealed that individuals with thalassemia have a significantly elevated total incidence rate of cancer, reaching 52%. Furthermore, the chance of developing lymphoma or leukemia was found to be 5.32 times greater in these patients. People with transfusion-dependent thalassemia have a 9.31-fold higher risk of developing hematological malignancies compared to people who do not require transfusions (119). Reported hematological tumors include hematological diseases such as acute lymphoblastic leukemia (ALL), acute myeloid leukemia (AML), acute promyelocytic leukemia (APL), chronic myeloid leukemia (CML), essential thrombocythaemia (ET), Hodgkin lymphoma (HL), multiple myeloma (MM), myeloproliferative neoplasm (MPN), non-Hodgkin lymphoma (NHL) and Diffuse large B cell lymphoma (DLBCL), etc (120–123). The increased tumor risk in patients with thalassemia may be related to oxidative damage secondary to iron accumulation, immune abnormalities, viral infections, IO, hydroxyurea use, and BM stimulation caused by chronic anemia (124, 125). Furthermore, thalassemia can manifest after the development of a tumor. The loss of α-globin gene clusters and the inactivation of somatic mutations in the trans-acting chromatin-associated protein ATRX usually lead to a significant decrease in α-globin gene expression (126). This type of acquired thalassemia is also seen in Hodgkin’s lymphoma, Juvenile Myelomonocytic Leukemia, and others (127, 128). However, the mechanism related to thalassemia and hematologic tumorigenesis is still unclear, which needs more research to reveal.

## 7 Other

Endothelial cell activation and dysfunction are confirmed in BT, mostly due to the inhibitory effect of ADMA on NO (nitric oxide) production and the accumulation of iron, which disrupts endothelial function (129–131). Endothelial cell apoptosis in the circulation involves the mitogen-activated protein kinases/Jun N-terminal Kinase (MAPK/JNK) signaling pathway (132). Currently, ongoing studies on BT endothelium are mostly centered around investigating the relationship between direct or indirect measures of IO (namely serum ferritin, transfusion burden, and MRI results) and outcome parameters (133). Furthermore, several research investigations have demonstrated an elevation in levels of adhesion molecules (intracellular adhesion molecule-1, slCAM-1, sVCAM-1, E-selectin, and P-selectin) and inflammatory factors (IL-6 and IL-1β) in individuals with thalassemia, along with an increase in tissue factor levels (134, 135). Long-term exposure to compromised erythropoiesis in the BM microenvironment was not directly considered. According to studies, atorvastatin, a widely used lipid-lowering drug in clinical practice, improves the functional impairment of BM EPC in the body by downregulating the p38 MAPK pathway. Moreover, NAC was also found to reduce ROS levels *in vitro* and *in vivo*, while repairing damaged BM ECs to effectively promote hematopoietic reconstruction (105, 106).

Macrophages within erythrocyte islands are central to the normal differentiation and development of erythrocytes (136). After being cultured with murine macrophages, it was discovered that the erythrocyte precursors extracted from BT BM were phagocytosed by the macrophages (137). Even while erythrocyte precursors derived from healthy persons were likewise phagocytosed, there was a significant rise in the activity of BT BM macrophages (137, 138). This may suggest that macrophage phagocytosis is enhanced in BT BM. Marrow adipose tissue(MAT), one of the main components of the BM stroma, plays a crucial role in maintaining hematopoietic function (139). However, the MAT was decreased in the BM of BT individuals with inefficient erythropoiesis, particularly in the BM fat fraction (BMFF) in red and yellow BM regions. This indicates that the transformation process of the BM was hindered (35, 140).

## 8 Conclusion

In summary, the review focused on the compromised BM microenvironment in patients with BT and the subsequent chain of events, which has an adverse impact on the patient. Hence, it is imperative to focus on the compromised BM microenvironment of patients, particularly IO, ROS, and bone resorption. Patients should get iron chelation therapy, antioxidant therapy, hypoglycemic therapy, and therapy to improve bone mineral density at an appropriate time.

## Author contributions

ZL: Funding acquisition, Methodology, Project administration, Writing – original draft. XY: Supervision, Writing – review & editing. JZ: Formal analysis, Supervision, Writing – original draft. JY: Conceptualization, Formal analysis, Writing – review & editing. JN: Conceptualization, Visualization, Writing – review & editing. YW: Funding acquisition, Project administration, Writing – review & editing.
